# Genome‐wide gain‐of‐function screening identifies EZH2 mediating resistance to PI3Kα inhibitors in oesophageal squamous cell carcinoma

**DOI:** 10.1002/ctm2.835

**Published:** 2022-05-23

**Authors:** Hui Xing, Mengshi Gao, Yuxiang Wang, Xu Zhang, Jiajie Shi, Xiang Wang, Xueling Liu, Qingyang Ma, Xiangyin Kong, Chunhao Yang, Jian Ding, Linghua Meng

**Affiliations:** ^1^ Division of Anti‐tumor Pharmacology, Shanghai Institute of Materia Medica Chinese Academy of Sciences Shanghai China; ^2^ School of Pharmaceutical Sciences University of Chinese Academy of Sciences Beijing China; ^3^ Key Laboratory of Tissue Microenvironment and Tumor, Shanghai Institute of Nutrition and Health Chinese Academy of Sciences Shanghai China; ^4^ Department of Medicinal Chemistry, Shanghai Institute of Materia Medica Chinese Academy of Sciences Shanghai China; ^5^ Division of Anti‐tumor Pharmacology, State Key Laboratory of Drug Research, Shanghai Institute of Materia Medica Chinese Academy of Sciences Shanghai China

**Keywords:** CDKN1A, cell cycle, CRISPR‐SAM, CYH33, ESCC, EZH2, PI3Kα inhibitor, resistance

## Abstract

Phosphoinositide‐3 kinase alpha (PI3Kα) has been confirmed to be a potential therapeutic target for esophageal squamous cell carcinoma (ESCC), while the potency of PI3Kα inhibitors is often attenuated by concurrent oncogenic signalling pathways. We performed genome‐wide gain‐of‐function screening with a CRISPR‐SAM library and identified enhancer of zeste homolog 2 (EZH2) rendering ESCC cells resistant to the PI3Kα inhibitor CYH33. Enhanced expression of EZH2 frequently occurs in ESCC and is related to poor prognosis. Overexpression of full‐length EZH2 but not methyltransferase‐deficient EZH2 conferred resistance to CYH33, while downregulating EZH2 expression restored sensitivity. EZH2 expression was negatively related to the activity of CYH33 against the proliferation of ESCC cell lines and patient‐derived cells. Transcriptomic analysis revealed that EZH2 abrogated CYH33‐mediated cell cycle regulation. EZH2 epigenetically suppressed the transcription of *CDKN1A*, promoting RB phosphorylation and cell cycle progression. Concurrently targeting EZH2 significantly potentiated CYH33 to inhibit the growth of ESCC cells and patient‐derived xenografts accompanied by enhanced cell cycle arrest. Taken together, our study demonstrated that an EZH2‐p21‐RB axis remodeled cell cycle regulation and rendered resistance to PI3Kα inhibitors in ESCC. Simultaneously targeting PI3Kα and EZH2 may provide an effective strategy for ESCC therapy with high expression of EZH2.

## Graphical abstract

- CRISPR‐SAM‐based gain‐of‐function screening identified the histone methyltransferase EZH2 rendering ESCC resistant to PI3Kα inhibitors.
- EZH2‐p21‐RB axis remodeled the cell cycle regulation and mediated the resistance.
- Simultaneously targeting PI3Kα and EZH2 may provide an effective strategy for the therapy of ESCC with high expression of EZH2.

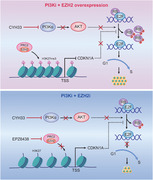

## 1 INTRODUCTION

Esophageal carcinoma (EC) has been a leading threat to human health, with a high incidence (604 000 new cases/year) and mortality (544 000 deaths/year) according to Global Cancer Statistics 2020.^1^ Esophageal squamous cell carcinoma (ESCC) represents the predominant subtype of all ECs in the world, especially in Asia and Africa.^2^, ^3^ Owing to the late diagnosis and limited therapy, the overall 5‐year survival rate of ESCC patients is less than 20%.^4^ Although immunotherapy targeting programmed cell death protein‐1/programmed cell death‐ligand 1 (PD‐1/PD‐L1) has emerged as the second‐line treatment for advanced ESCC,^5^, ^6^ the overall response rate is approximately 20%.^7^ ESCC is still an unmet medical need around the world.

Large‐scale next‐generation sequencing (NGS) has revealed frequent alterations in signalling pathways involving DNA damage repair, growth factor/receptor, cell cycle progression, etc., which provide potential therapeutic targets for ESCC.^8^, ^9^, ^10^, ^11^ Aberrantly activated phosphatidylinositol 3‐kinase (PI3K) pathway via amplification or mutation of *PIK3CA*, hyperactivation of RTKs, alteration of *AKT* or functional loss of *PTEN* occurs frequently in ESCC. Selective inhibition of PI3Kα significantly suppressed the growth of xenografts derived from human ESCC cells or patients.^12^, ^13^ Preliminary results from a phase II study of BKM120, which is a PI3K pan inhibitor, displayed promising efficacy and was tolerable as a monotherapy for pretreated advanced ESCC patients.^14^ Clinical trials with PI3Kα‐specific inhibitors, including alpelisib and CYH33, are undergoing ESCC therapy (NCT03292250, NCT01822613 and NCT03544905),^15^ while no results have been released to date. As PI3K sits in the centre of complex signalling pathways and the coexistence of multiple genetic alterations in ESCC, the efficacy of PI3Kα inhibitors is often attenuated by concurrent oncogenic signalling pathways. Although sporadic genes such as *KRAS*,^16^ *AXL*,^12^ *mTOR* ^17^ and *KMT2D* ^18^ have been verified to mediate resistance to PI3Kα inhibitors in various carcinomas, it would be desirable to screen on a whole‐genome scale and identify genes potentially rendering ESCC resistance to PI3Kα inhibitors with the aim of proposing strategies to improve efficacy. Recently, clustered regularly interspaced short palindromic repeats (CRISPR)‐based gene editing has provided a powerful tool for screening to discover genes mediating drug resistance on a genome‐wide scale.^19^

CYH33 is a novel and potent PI3Kα inhibitor discovered by our group.^20^ It is currently in phase I‐II clinical trials for the treatment of advanced solid tumours, including ESCC (NCT03544905, NCT04586335 and NCT05043922). The preliminary data from the phase I trial indicated that CYH33 possesses favourable pharmacokinetics and is well‐tolerated.^21^ Complete or partial response was observed in patients harbouring *PIK3CA* mutation when CYH33 was administered as a single agent.^22^ We previously found that CYH33 exhibited promising efficacy against ESCC tumour cells, cell‐derived xenografts (CDXs) and patient‐derived xenografts (PDXs),^13^, ^23^ while its activity is highly variable among ESCC cells. In an effort to identify genes mediating resistance to CYH33 in ESCC, CRISPR–Cas9‐mediated genome‐wide gain‐of‐function (GOF) screening was employed. Overexpression of enhancer of zeste homolog 2 (EZH2) rendered ESCC cells resistant to CYH33, which was associated with EZH2‐mediated cell cycle progression. Enhanced cell cycle arrest by the EZH2 inhibitor EPZ6438^24^ remarkably potentiated the efficacy of CYH33 against ESCC cells as well as PDXs. Thus, our study provides a mechanistic rationale for simultaneously targeting PI3Kα and EZH2 in ESCC.

## 2 MATERIALS AND METHODS

### 2.1 Compounds and reagents

CYH33 was provided by Shanghai HaiHe Biopharma Co., Ltd. (Shanghai, China). Alpelisib (Novartis, Cambridge, MA, USA) and EPZ6438 (Epizyme, Cambridge, MA, USA) were supplied by Selleck (Houston, TX, USA). GDC‐0077 (Genentech, San Francisco, CA, USA) was obtained from MedChemExpress (Monmouth Junciton, NJ, USA). Dimethyl sulfoxide (DMSO) and polybrene were purchased from Sigma‐Aldrich (St. Louis, MO, USA). Tween 80 and CMC‐Na were purchased from Sangon Biotech (Shanghai, China) and Sinopharm (Beijing, China), respectively. Compounds were dissolved in DMSO at 10 mM (CYH33, alpelisib and GDC‐0077) or 50 mM (EPZ6438) and then stored at −20℃. Stock solutions were diluted to the desired concentrations freshly in fetal bovine serum‐free medium before experiments. The final concentration of DMSO did not exceed .1% (v/v). For animal studies, compounds were dissolved in normal saline containing .5% Tween 80 (v/v) and .5% CMC‐Na (m/v).

### 2.2 Cell lines and cell culture

ESCC KYSE140, KYSE180, KYSE410, KYSE450 and KYSE510 cells were kindly provided by Dr. Hideaki Shimada (Department of Surgery, Toho University School of Medicine). TE1, TE5, TE6, TE8, TE10, TE11, TE14, TE15, T.T and OE19 cells were obtained from RIKEN Cell Bank (constructed by Dr. Nishihira, Tetsuro). HEK293T cells were provided by Dr. Jingyu Lang (Shanghai Institute of Nutrition and Health, CAS). All cell lines were analysed using short tandem repeat profiling^25^ for authentication by Genesky Bio‐Tech (Shanghai, China). Cells were incubated with the recommended medium in a humidified atmosphere containing 5% CO_2_ at 37°C.

### 2.3 Plasmids and lentivirus transfection

The human CRISPR three‐plasmid activation pooled library was a gift from Feng Zhang (#1000000057, Addgene, Watertown, MA, USA). Plasmids expressing human EZH2 and EZH2ΔSET *(del 617–738)* were constructed by VectorBuilder (Guangzhou, China). Human p21 was cloned and ligated into the expression vector pCDH‐CMV‐MCS‐EF2, which was obtained from Synbio Technologies (Suzhou, China). HEK293T cells were transfected with plasmids of interest, psPAX and pMD2.G at a ratio of 4:3:1 using Lipofectamine 2000 (Invitrogen, Carlsbad, CA, USA) as instructed by the manufacturer. The medium was replaced 6 h later and lentivirus‐containing supernatant was collected with a .45‐μm filter 48 h post‐transfection. Cells were infected with the filtered virus aided by 8 μg/ml polybrene and then selected with the indicated antibiotics.

### 2.4 CRISPR‐based gain‐of‐function screening and data analysis

The CRISPR‐synergistic activation mediator (SAM) system was employed to induce transcriptional activation at endogenous genomic loci.^26^ KYSE510 stably expressing dCas9‐VP64 and MS2‐p65‐HSF1 were transduced with the lentiviral‐pooled sgRNA library at low multiplicity of infection (MOI ≈ .3). Transduced cells were cultured with zeocin for 7 days and then treated with DMSO or CYH33 (1 μM). After 2 weeks of treatment, genomic DNA was extracted with a QIAamp DNA Blood Maxi Kit (QIAGEN, Duesseldorf, Germany). The sgRNA fragments were amplified by two‐step polymerase chain reaction (PCR) with the indicated primers (Table S1). The PCR products were sequenced and analysed by GENEWIZ (Jiangsu, China).^27^ In brief, PCR products were sequenced with the Illumina HiSeq Xten System (Illumina, San Diego, CA, USA). Sequencing reads were aligned to the sgRNA library sequences and analysed with count_spacers.py. The counts of sgRNA reads were normalised, and differential analysis among the samples was performed.

### 2.5 siRNA transfection

Custom siRNAs (Genepharma, Shanghai, China) were employed to transfect cells with the help of Lipofectamine RNAiMAX (Invitrogen, Carlsbad, CA, USA) following a previous study.^28^ The sequences of the siRNAs are listed in Table S2.

### 2.6 Cell proliferation and colony formation

The proliferation of ESCC cells was detected by a standard sulforhodamine B (SRB) assay.^29^ The proliferation of ESCC patient‐derived cells (PDCs) was determined using CellTiter‐Glo assay (Promega, Corporation, WI, USA) conducted by 3D Medicines (Shanghai, China). The growth inhibition was calculated as (OD_DMSO_‐OD_compound_)/(OD_DMSO_‐OD_d0_)×100%. The half maximal inhibitory concentration of cell growth (GI_50_) was computed by four parameter concentration–response curve fitting with SoftMaxPro (Molecular Devices, California, USA). Cells seeded in 6‐well plates were allowed to form colonies in the presence of the indicated compounds for 10 days. Colonies were fixed with methanol (Sinopharm Chemical Reagent Co., Ltd, Shanghai, China) and stained with .1% crystal violet (Sinopharm Chemical Reagent Co., Ltd, Shanghai, Transduced cells China). Images were recorded with a ChemiDoc Touch Imaging System (Bio‐Rad, Hercules, CA, USA). Colonies were quantified by ImageJ (NIH).

### 2.7 Tissue microarray and immunohistochemical staining

A total of 94 tumour tissues and 61 adjacent normal tissues derived from Chinese ESCC patients were employed for immunohistochemical (IHC) staining of EZH2 (ZuoCheng Bio Company, Shanghai, China). The staining was evaluated manually by a pathologist. A DM6 B microscope (Leica, Wetzlar, Germany) was applied for the observation of the stained slides.

### 2.8 RNA sequencing

RNA extraction and subsequent sequencing were conducted at Shanghai Majorbio Bio‐Pharm Technology Co., Ltd. (Shanghai, China). Gene Set Enrichment Analysis (GSEA, http://software.broadinstitute.org/gsea/index.jsp) was applied to analyse the biological pathways.

### 2.9 Western blotting

Cells were lysed and subjected to standard western blotting.^30^ The primary antibodies against EZH2 (#5246), p110α (#4249), pAKT‐Ser473 (#4060), AKT (#4691), pRB‐Ser780 (#3590), pRB‐Ser807/811 (#8516), RB (#9313), H3K27me3 (#9733), p21 (#2947) (Cell Signaling Technology, Danvers, MA, USA), Histone3 (#17168‐11‐AP), GAPDH (#60004‐1‐1G) (Proteintech Group, Chicago, IL, USA) and β‐actin (#A5441) (Sigma–Aldrich) were used at the recommended dilutions. Images were captured by a ChemiDoc Touch Imaging System (Bio‐Rad).

### 2.10 Reverse transcription‐polymerase chain reaction

Total RNA was extracted by a HiPure Total RNA Mini Kit (#R4111‐03, Magen, Shanghai, China) and cDNA was generated by reverse transcription using HiScript II Q Select RT SuperMix (#R233‐01, Vazyme, Nanjing, China). Quantitative polymerase chain reaction (PCR) was conducted using iQ SYBR Green Supermix (Bio‐Rad) with a real‐time PCR system (ABI VIIA7, ThermoFisher, Sunnyvale, CA, USA). The primers employed are listed in Table S3. The relative mRNA level was normalised to that of β‐actin.

### 2.11 Chromatin immunoprecipitation

Chromatin immunoprecipitation (ChIP) assays were performed with the SimpleChIP Plus Enzymatic Chromatin IP Kit (Cell Signaling Technology, Danvers, MA, USA) according to the procedures provided by the manufacturer. Antibodies against H3K27me3 (#9733) and normal rabbit IgG (#2729) were purchased from Cell Signaling Technology (Danvers, MA, USA). Immunoprecipitated DNAs were then quantified by quantitative PCR with the primers listed in Table S4.

### 2.12 Cell cycle analysis

The cell cycle distribution was measured as described in a previous study.^30^ Data were collected with a FACS Calibur Instrument (BD Biosciences, Franklin Lake, NJ, USA). FlowJo (TreeStar, Ashland, OR, USA) was employed to analyse the cell cycle distribution.

### 2.13 Animal studies

PDXs of ESCC (ES‐06‐0003 & ES‐06‐0016) were established and preserved by WuXi AppTec (Shanghai, China). All procedures were conducted according to the principles of the Association for Assessment and Accreditation of Laboratory Animal Care (AAALAC) and were approved by the Institutional Animal Care and Use Committee (IACUC) at WuXi AppTec. Female nu/nu athymic BALB/c mice aged 6–8 weeks were obtained from Shanghai Lingchang Biotechnology Co., Ltd. (Shanghai, China). Tumour sections were cut into pieces of approximately 30 mm^3^ and then transplanted into the right backs of mice. When the tumour volume reached approximately 150 mm^3^, the mice were randomised and orally administered vehicle control, CYH33 at 12.5 mg/kg, EPZ6438 at 100 mg/kg or the combination once a day. Tumour volume and body weight were measured twice a week. The tumour volume, relative tumour volume (RTV) and the treatment to control ratio (T/C) were calculated.^13^

### 2.14 Combination analysis

Cells were treated with CYH33 and/or EPZ6438 at a ratio of 1:5 for 5 days and proliferation was determined by SRB assay. The combination index (CI) was computed as described previously^29^ unless otherwise indicated. A synergistic, additive or antagonistic effect was defined when a CI value was less than 1, equivalent to 1 or greater than 1, respectively.

### 2.15 Statistical analysis

For all experiments, data were analysed by GraphPad Prism 7.0 (California, CA, USA) and shown as mean ± standard deviation (SD) from at least three independent experiments unless otherwise indicated. Statistical significance was determined by unpaired *t* test (with Welch's correction) between two groups and one‐way ANOVA for three or more groups.

## 3 RESULTS

### 3.1 A genome‐wide gain‐of‐function screening identified EZH2 mediating resistance to the PI3Kα inhibitor CYH33

**FIGURE 1:**
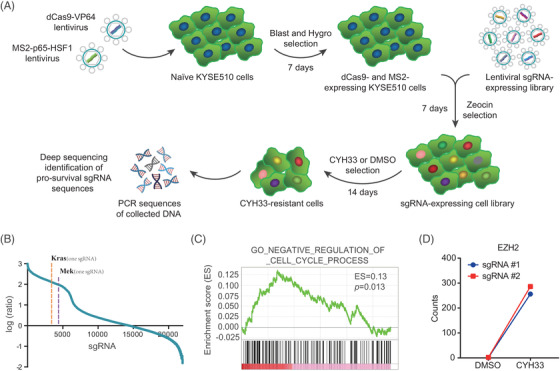
A genome‐wide gain‐of‐function screening identified enhancer of zeste homolog 2 (EZH2) mediating resistance to the PI3Kα inhibitor CYH33. (A) Schematic of CRISPR‐SAM‐based gain‐of‐function screening. The human CRISPR activation sgRNA library was transduced into KYSE510 cells stably expressing dCas9‐VP64 and MS2‐p65‐HSF1. sgRNA‐expressing cells selected by zeocin were exposed to CYH33 (1 μM) or dimethyl sulfoxide (DMSO) for 14 days. Genomic DNA was extracted from viable cells and sgRNA fragments were amplified by two‐step polymerase chain reaction (PCR). sgRNAs were identified by deep sequencing. Blast, blasticidin; Hygro, hygromycin. (B) Ratios of the sgRNA frequency in CYH33‐treated cells compared to that in control cells. sgRNAs targeting *KRAS* or *MEK* were enriched in cells treated with CYH33. (C) Gene Set Enrichment Analysis (GSEA) was performed using the ratios depicted in (B), and the enrichment of cell cycle regulation is presented. (D) The counts of sgRNA targeting *EZH2* in DMSO‐ and CYH33‐treated cells

To investigate genes associated with resistance to the PI3Kα inhibitor CYH33 (Figure S1A), we conducted an unbiased GOF screening in ESCC KYSE510 cells with a CRISPR‐SAM library consisting of 70 290 sgRNAs against 23 430 genes (Figure 1A). Deep sequencing was performed with DNA extracted from viable cells after treatment with DMSO or CYH33 (1 μM) for 14 days. As shown in Figure 1B, the ratio of the sgRNA frequency between CYH33‐ and DMSO‐treated cells was calculated and plotted. sgRNAs targeting *KRAS* and *MEK* were among the sgRNAs enriched in cells upon CYH33 treatment, which was consistent with previous reports that KRAS and MEK mediate resistance to PI3K inhibitors.^16^, ^31^, ^32^ To identify additional genes or pathways that might mediate resistance to CYH33 treatment, GSEA was performed with the ratios depicted in Figure 1B. Gene sets associated with MEK signalling, BMI and cell cycle regulation were significantly enriched (Figures 1C and S1B). Of particular note, the counts of sgRNAs targeting genes responsible for epigenetic modification (*PRMT2*, *PRMT3*, etc.) were increased in CYH33‐treated cells (Figure S1C), indicating that epigenetic regulation may mediate resistance to PI3Kα inhibitors. Among them, sgRNA targeting the histone methyltransferase EZH2 was remarkably enriched in CYH33‐treated cells, with ratios of 256 and 143, respectively, for two distinct sgRNAs (Figure 1D). Therefore, our primary screening suggested that overexpression of EZH2 may render ESCC cells resistant to PI3K inhibitors.

### 3.2 Overexpression of EZH2 was frequent and related to poor prognosis in ESCC

**FIGURE 2:**
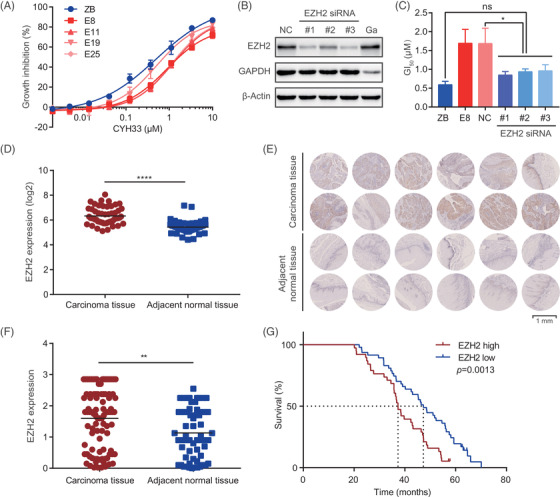
Overexpression of EZH2 was frequently found in esophageal squamous cell carcinoma (ESCC) and associated with poor prognosis. (A) Cells stably expressing EZH2 (E8, E11, E19 and E25) were generated by transfecting KYSE510 cells with dCas9‐VP64, MS2‐p65‐HSF1 and lentiviral EZH2‐targeted sgRNAs. ZB cells were infected with the empty vector. Cells were incubated with CYH33 for 72 h, and cell proliferation was determined by standard sulforhodamine B (SRB) assay (*n *= 3). (B) E8 cells were transfected with siRNAs targeting EZH2 for 48 h, and cell lysates were subjected to western blotting for the indicated proteins. NC, negative control; Ga, siRNA targeting GAPDH. (C) ZB cells, E8 cells or E8 cells transfected with siRNA targeting EZH2 (siEZH2 #1, #2 and #3) or negative control siRNA (NC) were treated with CYH33 for 72 h, and GI_50_s were determined by SRB assay. Data are presented as mean ± standard deviation (SD) from three independent experiments. Differences between the indicated groups were analysed by one‐way ANOVA with Tukey's multiple comparison test. Ns, no significance. *P < .05. (D) EZH2 mRNA data in ESCC tissues (*n *= 53) and adjacent normal tissues (*n *= 53) were retrieved from the Gene Expression Omnibus database (https://www.ncbi.nlm.nih.gov/geo/, GSE23400). Differences between groups were analysed by the Wilcoxon matched‐pairs signed rank test. ****P < .0001. (E, F) Representative images (E) and quantification (F) of immunohistochemical staining for EZH2 in tumour tissues (*n *= 94) and adjacent normal tissues (*n *= 61) from a cohort of Chinese ESCC patients. Scale bar, 1 mm. Differences between groups were analysed by unpaired *t* test. **P < .01. (G) Kaplan–Meier survival analysis of Chinese ESCC patients stratified by EZH2 expression (*P *= .0013 by log‐rank test)

EZH2 comprehensively participates in multiple cellular processes by epigenetically regulating gene transcription. Previous studies have shown that dysregulation of EZH2 induces resistance to chemotherapy,^33^, ^34^ targeted drugs^35^, ^36^ and immunotherapy.^37^, ^38^ EZH2 was associated with poor prognosis of advanced solid tumours, including gastric cancer^39^ and hepatocellular carcinoma.^40^ To confirm the resistance to CYH33 in ESCC mediated by EZH2, we generated KYSE510 cells stably expressing EZH2 by transfecting dCas‐VP64, MS2‐p65‐HSF1 and EZH2‐targeted sgRNA included in the CRISPR‐SAM library. Enhanced expression of EZH2 was observed in cells infected with the sgRNA (Figure S2A). CYH33 displayed reduced activity in EZH2‐overexpressing E8, E11, E19 and E25 cells in comparison with ZB cells transfected with the empty vector (Figures 2A and S2B). On the contrary, downregulating EZH2 by siRNAs restored the sensitivity to CYH33 in E8 cells (Figures 2B,C and S2C). These findings further confirmed that EZH2 mediated the resistance of ESCC cells to CYH33.

To explore the potential role of EZH2 in ESCC, EZH2 mRNA data were obtained from the Gene Expression Omnibus database (https://www.ncbi.nlm.nih.gov/geo/, GSE23400^41^). Elevated expression of EZH2 was found in 37.74% of ESCC tissues in comparison to normal counterparts with a fold change greater than 2 (Figure 2D). We further detected the expression of EZH2 in tumour tissues from 94 Chinese ESCC patients as well as 61 adjacent normal tissues by immunohistochemistry. Similarly, enhanced EZH2 expression at the protein level was found in ESCC tissues (Figure 2E,F). The cohort of ESCC patients was then grouped according to EZH2 expression and analysed with a Kaplan–Meier survival plot. The median survival times were 37.3 and 47.3 months in the EZH2‐high and EZH2‐low groups, respectively, suggesting significantly poorer survival in ESCC patients with higher expression of EZH2 (Figure 2G). Kaplan–Meier survival analyses of the patients stratified by EZH2 were further performed with consideration of age, sex, American Joint Committee on Cancer (AJCC) staging, TNM status and therapy. High expression of EZH2 was significantly associated with poor prognosis in ESCC patients regardless of the clinicopathologic features, although a few comparisons showed no significance due to the limited number of cases (Table S5). These results revealed that overexpression of EZH2 was frequent in ESCC and associated with poor prognosis.

### 3.3 Overexpression of EZH2 conferred resistance to CYH33 in ESCC cells

**FIGURE 3:**
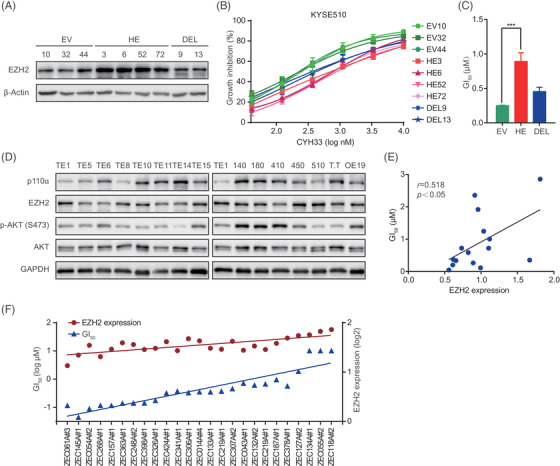
Overexpression of enhancer of zeste homolog 2 (EZH2) conferred resistance to CYH33 in esophageal squamous cell carcinoma (ESCC) cells. (A) A panel of monoclonal cells was generated by transfecting KYSE510 cells with empty vectors (EV), plasmids expressing human full‐length EZH2 (HE) or plasmids expressing truncated EZH2 without the SET domain (DEL). Protein levels of EZH2 were determined by western blotting. (B) The indicated cells in (A) were treated with CYH33 for 72 h. Cell proliferation was measured by standard sulforhodamine B (SRB) assay. Data shown are mean ± standard deviation (SD) from at least two independent experiments. (C) Average GI_50_s were calculated according to the data presented in (B) (*n* = 3 for EV, *n* = 4 for HE and *n* = 2 for DEL). Differences between two groups were determined by unpaired *t* test. ***P < .001. (D) Cell lysates of a panel of ESCC cells were subjected to western blotting with the indicated antibodies. (E) Pearson correlation analysis of EZH2 expression displayed in (D) and GI_50_s of CYH33 against the proliferation of ESCC cells. (F) ESCC patient‐derived cells were treated with serially diluted CYH33 for 72 h. The GI_50_s of CYH33 were determined by CellTiter‐Glo assay and the expression of EZH2 was analysed by RNA sequencing

As EZH2 is frequently overexpressed in ESCC, we further explored the impact of EZH2 on the antitumor activity of CYH33. Several clones of KYSE510 cells stably overexpressing EZH2 (HE) were established (Figure 3A). As shown in Figure 3B,C, enhanced EZH2 expression rendered KYSE510 cells less sensitive to CYH33, yielding an average GI_50_ of 0.89 μM in cells transfected with EZH2‐expressing plasmids (HE) compared to that of .25 μM in cells transfected with empty plasmids (EV). The Su(var)3‐9, enhancer of zeste and trithorax (SET) domain in the C‐terminus of EZH2 is crucial for its enzymatic activity, which provides binding sites for S‐adenosyl‐L‐methionine (SAM) and H3 lysine 27.^42^ Overexpression of EZH2 with deleted SET (DEL) had little effect on the antiproliferative activity of CYH33 (Figure 3B,C), indicating that the enzymatic activity of EZH2 was required to mediate resistance to CYH33. Overexpression of EZH2 also attenuated the activity of CYH33 to restrain the colonogenesis of ESCC cells (Figure S3A). Moreover, EZH2‐overexpressing HE6 cells were more resistant to alpelisib^43^ and GDC‐0077,^44^ which are known as potent PI3Kα‐selective inhibitors (Figure S3B), indicating that the resistance mediated by EZH2 was likely specific to PI3Kα inhibition rather than the potential off‐target effects.

To investigate the association between EZH2 expression and CYH33 activity in heterogeneous ESCC cells, we measured the protein levels of EZH2 and GI_50_s of CYH33 in multiple ESCC cell lines. EZH2 protein was positively correlated with the GI_50_s of CYH33, yielding a correlation coefficient of .518 (Figure 3D,E). A similar correlation was detected between the mRNA levels of *EZH2* obtained from cBioPortal (http://www.cbioportal.org) and the GI_50_s of CYH33 in the same panel of ESCC cell lines (*r *= .616) (Figure S3C). Meanwhile, no significant correlation was observed between CYH33 activity and p110α expression or phosphorylated AKT in ESCC cells (Figure S3D). Similar experiments were performed in 25 ESCC PDC lines. Most PDCs were sensitive to CYH33 with GI_50_s less than 1 μM, and cells with higher expression of EZH2 were more resistant to CYH33 (*r *= .835) (Figures 3F and S3E). Taken together, overexpression of EZH2 circumvented the activity of CYH33 in ESCC cells.

### 3.4 Epigenetic repression of *CDKN1A* by EZH2 attenuated the activity of CYH33 against ESCC

**FIGURE 4:**
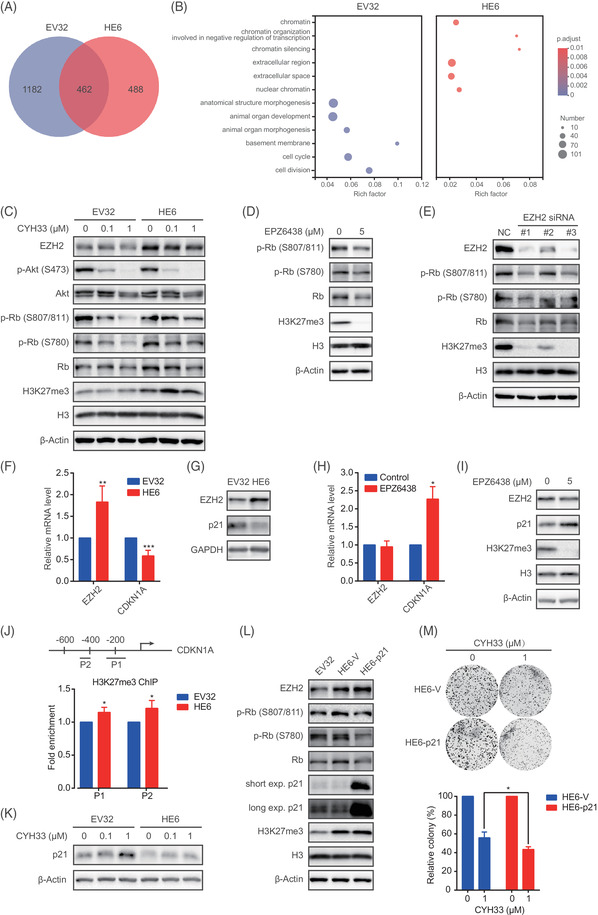
Epigenetic repression of *CDKN1A* by enhancer of zeste homolog 2 (EZH2) attenuated the activity of CYH33 against esophageal squamous cell carcinoma (ESCC). (A) HE6 cells stably expressing EZH2 or EV32 cells transfected with the empty vector were treated with CYH33 (1 μM) for 24 h, and RNA sequencing was performed. Differentially expressed genes (DEGs) identified with |fold change| ≥ 2 and Benjamini & Hochberg adjusted *P *< .05 upon CYH33 treatment were plotted as a Venn diagram. (B) Gene Ontology enrichment of the DEGs (1182 in EV32 cells and 488 in HE6 cells). The top six enriched gene sets in EV32 or HE6 cells are presented as a bubble chart. (C) EV32 or HE6 cells were treated with CYH33 at the indicated concentrations for 24 h, and cell lysates were subjected to western blotting with the indicated antibodies. (D) HE6 cells were treated with EPZ6438 (5 μM) for 4 days and cell lysates were subjected to western blotting for the indicated proteins. (E) HE6 cells were transfected with siRNAs targeting EZH2 (#1, #2 and #3) or negative control siRNA (NC) for 5 days, and cell lysates were subjected to western blotting with the indicated antibodies. (F, G) mRNA levels of the indicated genes were measured by quantitative polymerase chain reaction (PCR) (F), and protein levels were determined by western blotting (G) in EV32 and HE6 cells (*n* = 3). (H, I) HE6 cells were treated with EPZ6438 (5 μM) for 4 days. The mRNA levels of the indicated genes were measured by quantitative PCR (H), and the protein levels were determined by western blotting (I) (*n* = 3). (J) Fold enrichment of H3K27me3 in the promoter region of *CDKN1A* in EV32 or HE6 cells (*bottom*). The diagram shows the positions (P1: ‐274∼ ‐138; P2: ‐484∼ ‐399) of the primers used at the *CDKN1A* promoter (*top*). (K) EV32 and HE6 cells were treated with CYH33 at the indicated concentrations for 24 h. Protein levels of p21 were determined by western blotting. (L) HE6 cells were transfected with p21‐expressing plasmids (HE6‐p21) or empty vectors (HE6‐V), respectively. Cell lysates of EV32, HE6‐V or HE6‐p21 cells were subjected to western blotting with the indicated antibodies. (M) Colony formation assay of HE6‐V and HE6‐p21 cells treated with CYH33 (1 μM) for 10 days (*top*) and quantitation by ImageJ (*bottom*) (*n* = 3). Data shown are mean ± standard deviation (SD) or representatives from at least three independent experiments. Differences between two groups were analysed by unpaired *t* test. *P < .05, **P <.01 and ***P < .001

To explore the mechanism of EZH2‐mediated resistance to CYH33 in ESCC, RNA sequencing was performed to monitor the transcriptomic response upon CYH33 treatment in HE6 cells overexpressing EZH2 and EV32 cells transfected with the vector control. Differentially expressed genes (DEGs) were recognised with a fold change greater than 2 and a *P* value lower than .05, which were plotted as a Venn diagram. As shown in Figure 4A, a total of 1644 DEGs were found in EV32 cells after CYH33 treatment, whereas 950 DEGs were detected in HE6 cells. Although 462 DEGs were found in both EV32 and HE6 cells, forced expression of EZH2 reshaped the gene expression pattern of ESCC cells in response to CYH33. Gene Ontology enrichment analysis was performed to identify the biological significance of the DEGs distinctive in EV32 cells (1182 genes) or HE6 cells (488 genes). The top six enriched gene sets in EV32 or HE6 cells were visualised as a bubble chart (Figure 4B). Gene sets involved in the cell cycle and cell division were enriched in EV32 cells but not in HE6 cells, suggesting that high expression of EZH2 may abrogate the regulation of CYH33 in cell cycle progression. To test this hypothesis, we examined retinoblastoma protein (RB), which serves as a gatekeeper of the G1/S phase transition.^45^ As shown in Figure 4C, increased RB phosphorylation at serine 807/811 and serine 780 was observed in HE6 cells compared to EV32 cells. Moreover, although CYH33 treatment for 24 h significantly inhibited the phosphorylation of RB in EV32 cells, this effect failed to be achieved in HE6 cells, suggesting that the overexpression of EZH2 maintained active cell cycle progression in ESCC cells. Accordingly, treatment of HE6 cells with the SAM‐competitive EZH2 inhibitor EPZ6438 significantly decreased the phosphorylation of RB and trimethylation of histone 3 at lysine 27 (H3K27me3) (Figures 4D and S4A). Similarly, knockdown of EZH2 by specific siRNAs resulted in reduced phosphorylated RB (Figure 4E). To understand how EZH2 regulates RB phosphorylation, we surveyed the possible transcriptional targets of EZH2 according to available databases and previous studies. Cyclin‐dependent kinase inhibitors (CKIs) acting as negative regulators of cell cycle progression have emerged as potential targets of EZH2.^46^, ^47^, ^48^ We measured the mRNA level of a panel of CKIs in EV32 and HE6 cells. Overexpression of EZH2 in HE6 cells decreased the expression of *CDKN1A, CDKN2A*, *CDKN2B*, *CDKN2C* and *CDKN2D* (Figure S4B). *CDKN1A* was selected for further investigation because of its critical role in the G1/S transition and significant reduction upon EZH2 overexpression. Forced expression of EZH2 in HE6 cells led to a reduction in *CDKN1A* (p21) mRNA and protein (Figure 4F,G). Conversely, induction of *CDKN1A* (p21) mRNA and protein was observed in HE6 cells exposed to EPZ6438 for 5 days (Figure 4H,I). Meanwhile, p21 was inversely correlated with EZH2 expression in a panel of KYSE510‐derived monoclonal cells with forced expression of EZH2 (*r* = −.642) and a panel of ESCC cell lines (*r* = −.481) (Figure S4C,D). These findings indicated that EZH2 might suppress the transcription of *CDKN1A* in ESCC cells. Trimethylation of histone 3 at lysine 27 by EZH2 leads to the transcriptional repression of EZH2‐targeted genes. Chromatin immunoprecipitation was performed with an antibody against H3K27me3 followed by PCR to amplify the promoter region of *CDKN1A* in EV32 and HE6 cells. As shown in Figure 4J, H3K27me3 was enriched in the promoter region of *CDKN1A* in HE6 cells with elevated expression of EZH2, demonstrating that EZH2 may silence *CDKN1A* expression by H3K27 trimethylation. Moreover, forced expression of EZH2 in HE6 cells abrogated the induction of p21 by CYH33 treatment (Figure 4K). To investigate the role of p21 in EZH2‐mediated cell cycle regulation, HE6 cells were transfected with p21‐expressing plasmids. As shown in Figure 4L, the increased phosphorylated RB induced by EZH2 in HE6 cells was abrogated by overexpression of p21. Furthermore, overexpression of p21 potentiated the inhibition of clonogenesis by CYH33 in HE6 cells (Figure 4M). Consistently, counts of *CDKN1A* targeting sgRNAs were markedly decreased in CYH33‐treated cells with ratios of .0087 and .3106 in the CRISPR‐SAM‐based screening (Figure S4E), further indicating that overexpression of p21 rendered ESCC cells sensitive to CYH33. These findings suggested that an EZH2‐p21‐RB axis mediated resistance to CYH33 in ESCC cells.

### 3.5 Simultaneously inhibiting PI3Kα and EZH2 displayed synergistic activity against ESCC cells

**FIGURE 5:**
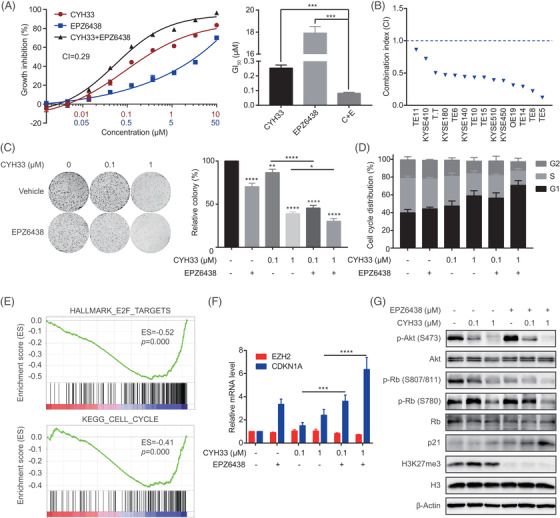
The concomitant inhibition of PI3Kα and enhancer of zeste homolog 2 (EZH2) displayed synergistic activity against esophageal squamous cell carcinoma (ESCC) cells. (A) HE6 cells were treated with CYH33 and EPZ6438 alone or concurrently for 5 days. Cell proliferation (*left*) and GI_50_s (*right*) were detected by standard sulforhodamine B (SRB) assay, and the combination index (CI) was determined by CalcuSyn software (*n* = 3). (B) CI values in a panel of ESCC cell lines were assessed by SRB assay (*n* = 3). (C) Colony formation assay of HE6 cells incubated with the indicated compounds for 10 days (*left*) and quantitation by ImageJ (*right*) (*n* = 3). (D) HE6 cells were exposed to EPZ6438 (5 μM) or dimethyl sulfoxide (DMSO) for 48 h and then concurrently with CYH33 at the indicated concentrations for 24 h. Cell cycle distribution was analysed by flow cytometry (*n* = 3). (E) Gene Set Enrichment Analysis (GSEA) was applied in cells treated with CYH33 alone or concurrently with EPZ6438. Downregulation of “HALLMARK_E2F_TARGETS” and “KEGG_CELL_CYCLE” in cells upon concurrent treatment with CYH33 (1 μM) and EPZ6438 (5 μM) is displayed. (F, G) HE6 cells were exposed to EPZ6438 (5 μM) or DMSO for 48 h and then concurrently with CYH33 at the indicated concentrations for 24 h. Total RNA was extracted for quantitative PCR (F) (*n* = 4), and cell lysates were subjected to western blotting with the indicated antibodies (G) (*n* = 3). Data are presented as mean ± SD or representatives from at least three independent experiments. Differences between two groups were analysed by unpaired *t* test. *P < .05, **P < .01, ***P < .001 and ****P < .0001

Given that overexpression of EZH2 rendered ESCC cells resistant, we sought to test whether inhibiting EZH2 could sensitise ESCC cells to CYH33. To this end, we treated HE6 cells with CYH33 or concurrently with EPZ6438 for 5 days. EPZ6438 potentiated the antiproliferative activity of CYH33 in HE6 cells with a CI value of .29 (Figure 5A). The combinatorial efficacy of CYH33 and EPZ6438 was then assessed in a panel of ESCC cell lines. A synergistic effect was detected in most tested cell lines with CI values less than 1 (Figure 5B). It should be noted that antagonism (CI = 1.5) was observed in RB‐deficient TE1 cells (Figure S5A), which supported our findings that EZH2 regulated cell cycle progression via the p21‐RB axis. Combinatorial effects of EPZ6438 and the FDA‐approved PI3Kα inhibitor alpelisib were also evaluated, yielding CI values of .25 and .26 in KYSE510 and KYSE180 cells with relatively high expression of EZH2, respectively (Figure S5B). The synergism was further verified by enhanced suppression of colony formation in ESCC cells concurrently treated with CYH33 and EPZ6438 compared to those treated with CYH33 alone (Figures 5C and S5C). Meanwhile, EPZ6438 slightly enhanced the effect of CYH33 on colonogenesis in KYSE410 and TE11 cells, which expressed lower levels of EZH2 (Figure S5C). These results suggested the rationale of combining the EZH2 inhibitor and PI3K inhibitor in EZH2‐overexpressing ESCC. Consistent with the regulation of EZH2 in the G1/S transition, the combination of CYH33 and EPZ6438 induced elevated G1 phase arrest compared to the monotherapy (Figures 5D and S5D). Moreover, RNA sequencing and subsequent GSEA revealed that the gene sets “HALLMAR_E2F_TARGETS” and “KEGG_CELL CYCLE” were remarkably downregulated in cells concurrently treated with CYH33 and EPZ6438 in comparison with those treated with CYH33 alone (Figure 5E). Several CKIs consisting of *CDKN1A* were included in the gene sets and found to be upregulated in the cells upon treatment with CYH33 and EPZ6438 (Figure S5E). Accordingly, the combined treatment induced the transcription of *CDKN1A*, further enhanced the expression of p21 and attenuated the phosphorylation of RB in ESCC cells (Figure 5F,G). Taken together, the results showed that the PI3Kα inhibitor combined with the EZH2 inhibitor synergistically suppressed the proliferation of ESCC cells by transcriptionally activating *CDKN1A* and inhibiting RB phosphorylation.

### 3.6 EPZ6438 potentiated CYH33 to inhibit the growth of ESCC PDXs

**FIGURE 6:**
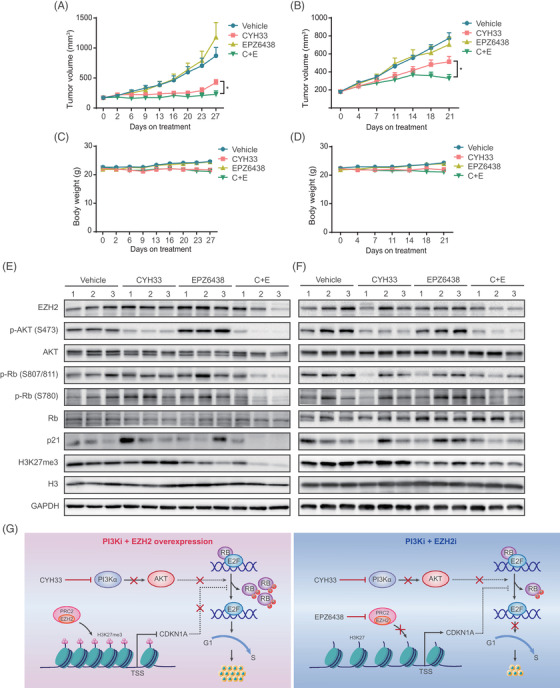
EPZ6438 potentiated CYH33 to inhibit the growth of esophageal squamous cell carcinoma (ESCC) patient‐derived xenografts (PDXs). (A‐D) Randomly grouped BALB/c nude mice bearing ESCC PDX ES‐06‐0003 (A, C) or ES‐06‐0016 (B, D) were orally administered vehicle control (*n* = 12), CYH33 (12.5 mg/kg, once a day), EPZ6438 (100 mg/kg, once a day) or the combination (*n *= 6). Tumour volume (A, B) and body weight (C, D) were measured twice per week, and the treatment to control ratios (T/C) were calculated at the end of treatment. Data presented are mean ± SEM. Differences between the indicated two groups were assessed by unpaired *t* test. *P <.05. (E, F) Tumour tissues collected from mice bearing ESCC PDX ES‐06‐0003 (E) or ES‐06‐0016 (F) at the end of the experiment were lysed for western boltting to detect the indicated proteins. (G) Schematic model of resistance to the PI3Kα inhibitor CYH33 driven by EZH2‐mediated epigenetic repression of *CDKN1A* and activation of cell cycle progression in ESCC cells (*left*). Inhibiting enhancer of zeste homolog 2 (EZH2) by EPZ6438 potentiated the activity of CYH33 against ESCC by releasing *CDKN1A* transcription and inhibiting RB phosphorylation as well as G1 phase arrest (*right*)

The combination of CYH33 and EPZ6438 in vivo was then evaluated in mice bearing PDXs derived from ESCC patients. ES‐06‐0003 harbouring amplification and mutation (E545K) in *PIK3CA* as well as amplification in *EZH2* was derived from a recurrent ESCC patient who was treated with radiotherapy before surgery. As shown in Figures 6A and S6A, CYH33 (12.5 mg/kg) significantly attenuated the growth of ES‐06‐0003, yielding a T/C value of 49.08%, while EPZ6438 at 100 mg/kg failed to restrain the growth of PDXs. The combination of these two drugs significantly elevated the efficacy of monotherapy (T/C value of 26.68%). ES‐06‐0016 was derived from a heavy smoker ESCC patient with amplified *EZH2* and wild‐type *PIK3CA*. Daily treatment with CYH33 (12.5 mg/kg) displayed mild activity against tumour growth (T/C value of 66.60%). EPZ6438 alone slightly inhibited tumour growth (T/C value of 91.35%). The combination of CYH33 and EPZ6438 potently suppressed tumour growth, achieving a T/C value of 42.63% (Figures 6B and S6B). No significant loss of body weight was detected in mice during the treatment (Figure 6C,D), indicating good tolerance to the combination therapy.

Tumour tissues were collected at the end of the experiments, and western blotting was performed. As shown in Figure 6E,F, CYH33 significantly inhibited the phosphorylation of Akt, while EPZ6438 reduced the methylation of H3 (H3K27me3), indicating potent inhibition of their respective targets in tumor tissues. Concurrent treatment with CYH33 and EPZ6438 displayed enhanced activity to attenuate the phosphorylation of RB, supporting that the synergistic activity of the combination resulted from cell cycle arrest. However, the expression of p21 was highly variable among the individual mice in the same treatment group and failed to accumulate after the combinatorial treatment. The inconsistency with the observation in ESCC cells might result from the more complicated context in ESCC PDXs and the possible regulation of EZH2 on other CKIs, which deserves further investigation. Nevertheless, the findings showed that EPZ6438 enhanced the efficacy of CYH33 against the growth of ESCC PDXs by suppressing RB phosphorylation and cell cycle progression.

## 4 DISCUSSION

Hyperactivation of *PIK3CA* is frequently found in ESCC, while the efficacy of PI3Kα inhibitors  was mild and variable as monotherapy. In this study, we found that overexpression of EZH2 mediated resistance to PI3Kα inhibitors in ESCC by unbiased GOF screening with a CRISPR‐SAM library. EZH2 was revealed to enhance the enrichment of H3K27me3 in the promoter region of *CDKN1A* and suppress its transcription and expression, which promoted RB phosphorylation and cell cycle progression. Thus, we proposed an EZH2‐CKIs‐Rb axis to remodel cell cycle regulation and render ESCC cells resistant to PI3Kα inhibitors. Moreover, concurrently targeting EZH2 exhibited a synergistic effect with CYH33 against ESCC cells as well as PDXs, which provided the mechanistic rationale for combining CYH33 with EZH2 inhibitors for ESCC treatment (Figure 6G).

CRISPR‐based high‐throughput screening is powerful for identifying tumour‐driving or synergistic lethal genes with the aim of overcoming drug resistance.^49^, ^50^, ^51^ Consistent with the heterogeneity of ESCC, we found that multiple oncogenic pathways might mediate resistance to PI3K inhibitors. Among them, the KRAS/MEK pathway has been proven to abrogate the activity of PI3K inhibitors against breast and lung cancer.^16^, ^31^, ^32^ Although CRISPR‐based loss‐of‐function (LOF) screening is frequently employed to identify essential genes that potentially lead to drug resistance or synergistic lethality, the identified genes are limited to the preexpressing genes in the cells. The CRISPR‐based SAM system allows transcriptional activation on a whole‐genome scale and induces the expression of genes that are silenced in the cells. Thus, this GOF genome is more likely to mimic the heterogeneity of ESCC. In particular, we found multiple sgRNAs targeting EZH2 enriched in CYH33‐treated cells, indicating that epigenetic regulation may affect the activity of PI3Kα inhibitors. In contrast to frequent mutation in non‐Hodgkin's lymphoma (∼22%),^52^ alteration of EZH2 is rare, and its role remains largely unknown in ESCC. A recent study extensively profiled the DNA methylome in ESCC and found that hypermethylated regions were enriched in those recognised by the polycomb repressive complex (EZH2/SUZ12), indicating the potential role of EZH2 in ESCC.^53^ Therefore, CRISPR‐based GOF screening is helpful to identify genes that are important for cancer therapy without the appearance of genomic alterations.

EZH2 is involved in global transcriptional repression, which plays an important role in cancer progression by silencing tumor‐suppressor genes. EZH2 is a validated therapeutic target for some types of lymphoma, such as diffuse large B‐cell lymphoma, in which activating mutation of EZH2 is frequently detected. Although such genetic alteration is rare in advanced solid tumors, high expression of EZH2 was associated with the progression of prostate cancer,^54^, ^55^ breast cancer^56^ and hepatocellular carcinoma.^40^ Sporadic studies have reported that overexpression of EZH2 is correlated with aggressiveness and poor prognosis in ESCC.^57^ Indeed, we found that the expression of EZH2 was elevated in ESCC compared to normal tissues, which was positively correlated with the shorter overall survival time by analysing publicly available data or a cohort of Chinese ESCC patients. EZH2 was identified to confer resistance of ESCC cells to PI3Kα inhibitors, which was further confirmed by the negative correlation of CYH33 activity and EZH2 expression in multiple ECSS cell lines and patient‐derived cells. Moreover, overexpression of full‐length EZH2 but not the truncated EZH2 (ΔSET) rendered ESCC cells resistant to CYH33, suggesting that the trimethylation of histone 3 at lysine 27 mediated by EZH2 and subsequent global transcriptional regulation is required for this process. We revealed that the EZH2‐CKI‐Rb axis might contribute to resistance to CYH33. Overexpression of EZH2 enriched H3K27me3 at the promoter region of *CDKNIA* and reduced transcription of the gene as well as increased phosphorylated RB, which was consistent with the abrogation of the cell cycle regulation mediated by CYH33 in EZH2‐overexpressing HE6 cells. We also found that EZH2 induced transcriptional repression of a number of CKIs, including *CDKN2A*, *CDKN2B*, *CDKN2C* and *CDKN2D*, which have been proven to be potential targets of EZH2.^58^, ^59^ It deserves further investigation to elucidate whether different CKIs mediate EZH2‐induced resistance to PI3Kα inhibitors under different circumstances.

Consistent with the observation that the activation of EZH2 mediated resistance to PI3Kα inhibitors in ESCC cells, simultaneously targeting EZH2 significantly improved the efficacy of CYH33 against ESCC cells and ESCC PDXs, accompanied by reduced RB phosphorylation and enhanced G1 phase arrest. Similarly, inhibiting EZH2 has been reported to enhance the sensitivity of the pan‐PI3K inhibitor copanlisib in lung cancer cells harbouring mutated or amplified *PIK3CA* when the manuscript was in preparation.^60^ The crosstalk between PI3K signaling and epigenetic regulation has been increasingly recognised. PI3Kα inhibition was found to enhance *ER* transcription by dephosphorylating and releasing KMT2D activity in breast cancer cells,^18^, ^61^ which leads to a rationale for targeting the epigenome and PI3K signalling. BET inhibitors and PI3K inhibitors have been shown to display synergism in neuroblastoma^62^ and B‐cell lymphoma.^63^ To our knowledge, we report for the first time the experimental therapy of ESCC by concurrently targeting EZH2 and PI3Kα. EZH2 inhibitors may improve the clinical efficacy of CYH33 in ESCC patients highly expressing EZH2. On the other hand, EZH2 inhibitors are yet confined to haematological malignancies with GOF mutations in *EZH2* ^64^ due to their limited efficacy in solid tumors.^65^ Our findings suggest a strategy of synthetic lethality that may extend the utilisation of EZH2 inhibitors in the treatment of solid tumours.

In summary, CRISPR–Cas9‐mediated genome‐wide GOF screening identified that EZH2 rendered ESCC cells resistant to the PI3Kα inhibitor CYH33, which was associated with EZH2‐mediated cell cycle progression via the p21‐RB axis. The EZH2 inhibitor EPZ6438 significantly potentiated the activity of CYH33 against ESCC cells as well as PDXs accompanied by enhanced inhibition of cell cycle progression. Thus, our study provided a mechanistic rationale to concurrently target PI3Kα and EZH2 in ESCC with high expression of EZH2.

## CONFLICT OF INTEREST

Jian Ding is the Chairman of Haihe Biopharma Pharmaceutical Co., Ltd. No conflict of interest is disclosed for the rest of the authors.

## Supporting information

Supporting Information
