# Supplementary material for: Genome‐wide gain‐of‐function screening identifies EZH2 mediating resistance to PI3Kα inhibitors in oesophageal squamous cell carcinoma
Source: Clin Transl Med. 2022 May 23;12(5):e835. doi: 10.1002/ctm2.835 (PMC9126361; doi:10.1002/ctm2.835)
Supplement: Supplementary file 1 — Supporting Information [file CTM2-12-e835-s001.docx]

**SUPPORTING INFORMATION**

**
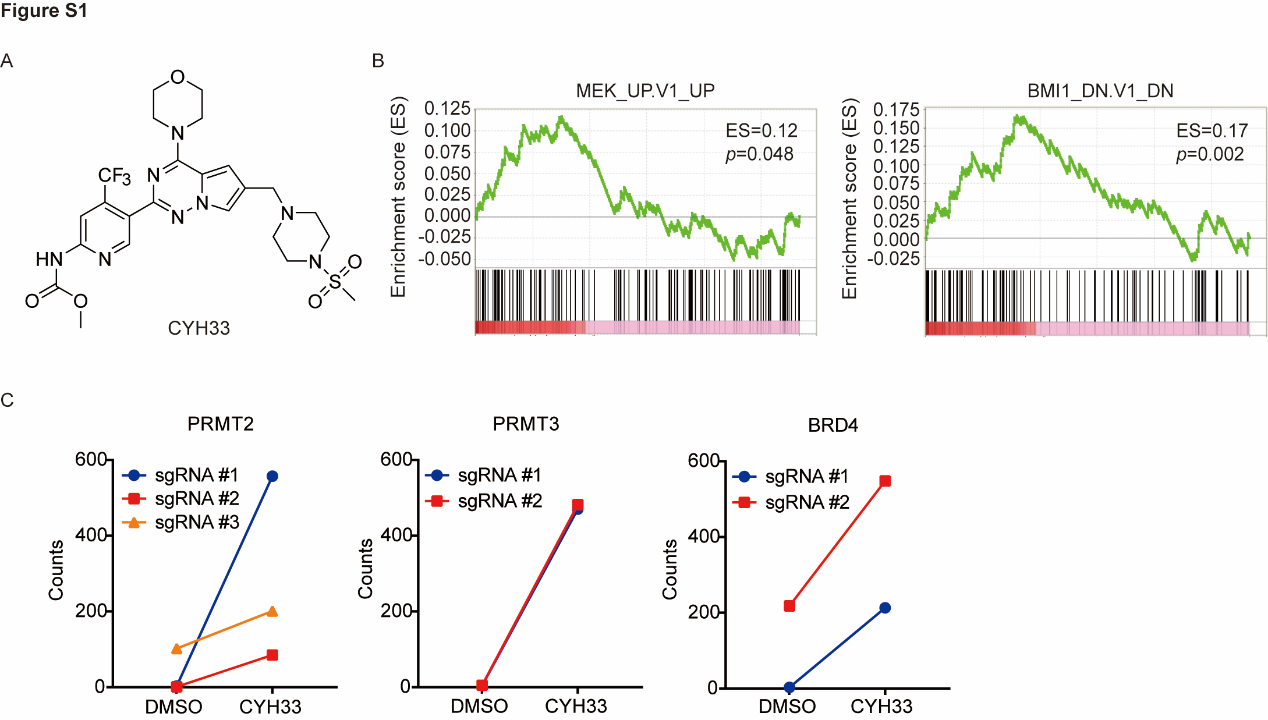
**

**FIGURE S1** A genome-wide gain-of-function screening identified MEK signaling and epigenetic regulation mediating resistance to the PI3Kα inhibitor CYH33. (A) Structure of the PI3Kα inhibitor CYH33. (B) GSEA was performed with ratios depicted in Figure 1B and the enrichment plots of MEK signaling and BMI were presented. (C) The counts of sgRNAs targeting genes in charge of epigenetic modification in DMSO- and CYH33-treated cells.


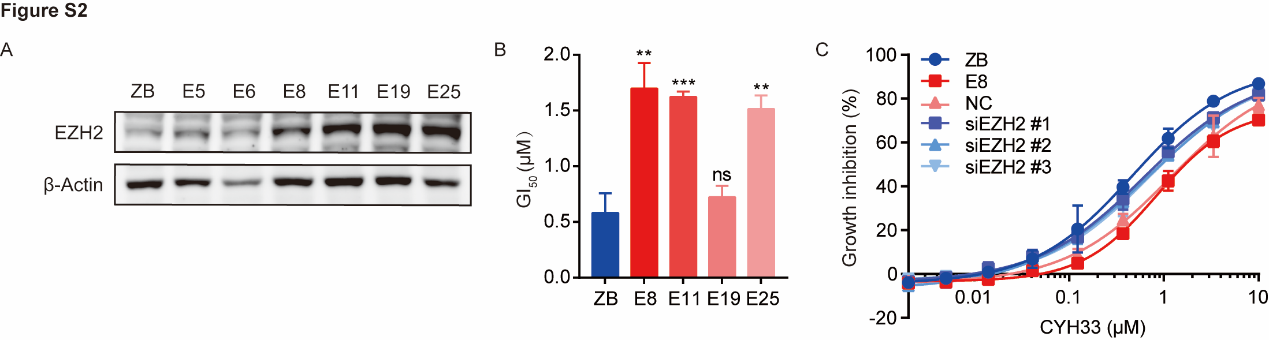


**FIGURE S2** EZH2 mediated resistance to CYH33 in ESCC cells. (A) KYSE510 cells stably expressing dCas9-VP64 and MS2-p65-HSF1 were transfected with lentiviral EZH2-targeted sgRNAs or empty vectors (ZB). Monoclonal cells were selected and lysed for Western blot with the indicated antibodies. (B) Cells stably expressing EZH2 (E8, E11, E19 and E25) were incubated with CYH33 for 72 h and GI_50_s were determined by SRB assay. (C) ZB cells, E8 cells or E8 cells transfected with siRNA targeting EZH2 (siEZH2 #1, #2 and #3) or negative control siRNA (NC) were treated with CYH33 for 72 h and cell proliferation was determined by SRB assay. Data presented are mean ± SD from three independent experiments. Difference between groups were analyzed by unpaired *t* test. ns, no significance. ***p* < 0.01 and *** *p* < 0.001.


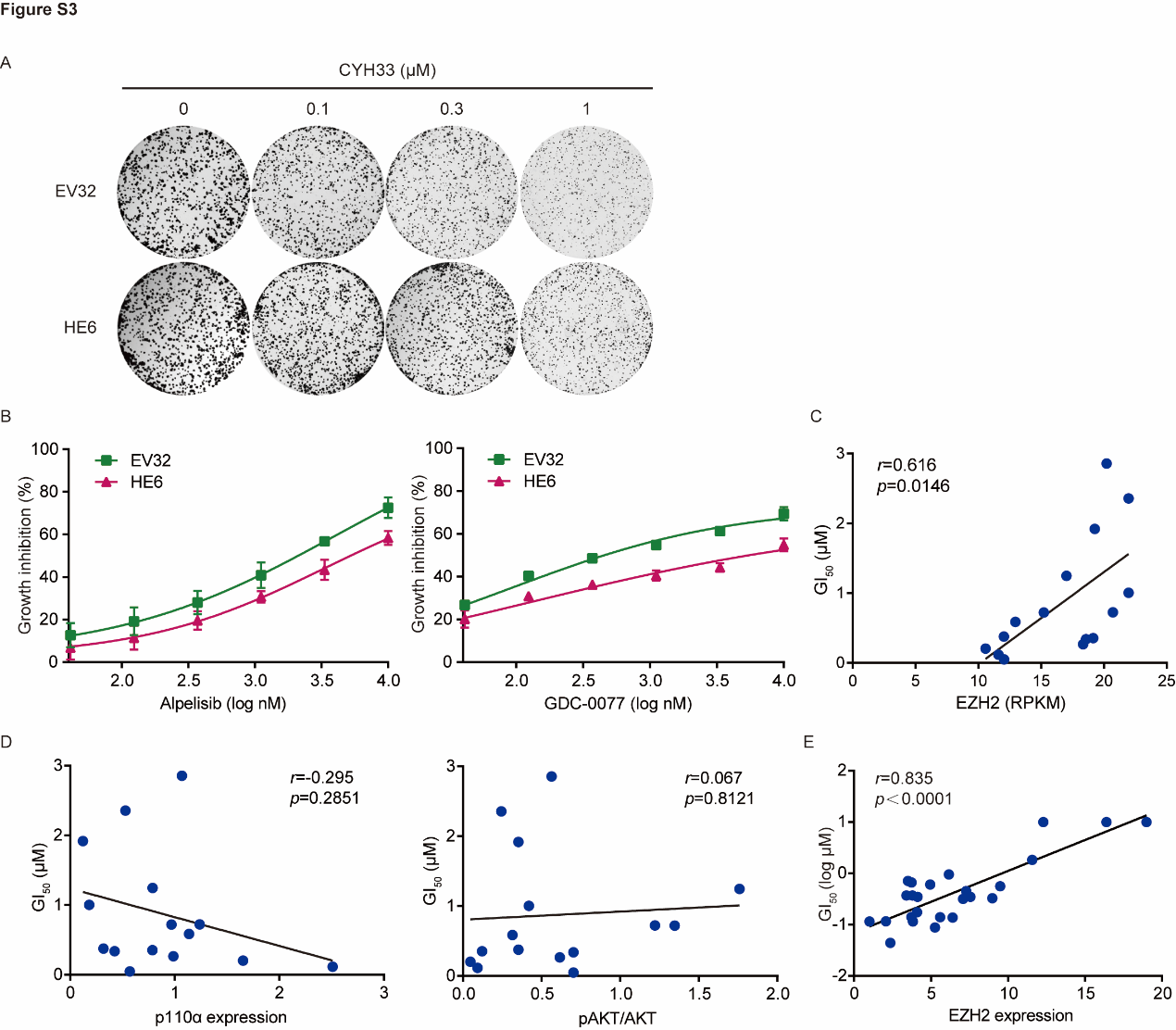


**FIGURE S3** Overexpression of EZH2 conferred resistance to PI3Kα inhibitors in ESCC cells. (A) Colony formation assay of EV32 and HE6 cells upon treatment of CYH33 at the indicated concentrations for 10 d. (B) EV32 and HE6 cells were treated with the PI3Kα inhibitor alpelisib (*left*) or GDC-0077 (*right*) for 72 h and cell proliferation was detected by SRB assay (*n* = 3). (C) Pearson correlation analysis between mRNA levels of EZH2 obtained from cBioPortal (<http://www>.cbioportal.org) and GI_50_s of CYH33 analyzed by SRB assay in a panel of ESCC cell lines. RPKM, Reads Per Kilobase per Million mapped reads. (D) Pearson correlation analysis of p110α expression (*left*) or phosphorylated AKT (*right*) and GI_50_s of CYH33 in ESCC cell lines presented in Figure 3D. (E) Pearson correlation analysis of EZH2 expression and GI_50_s of CYH33 in ESCC patient-derived cells presented in Figure 3F.


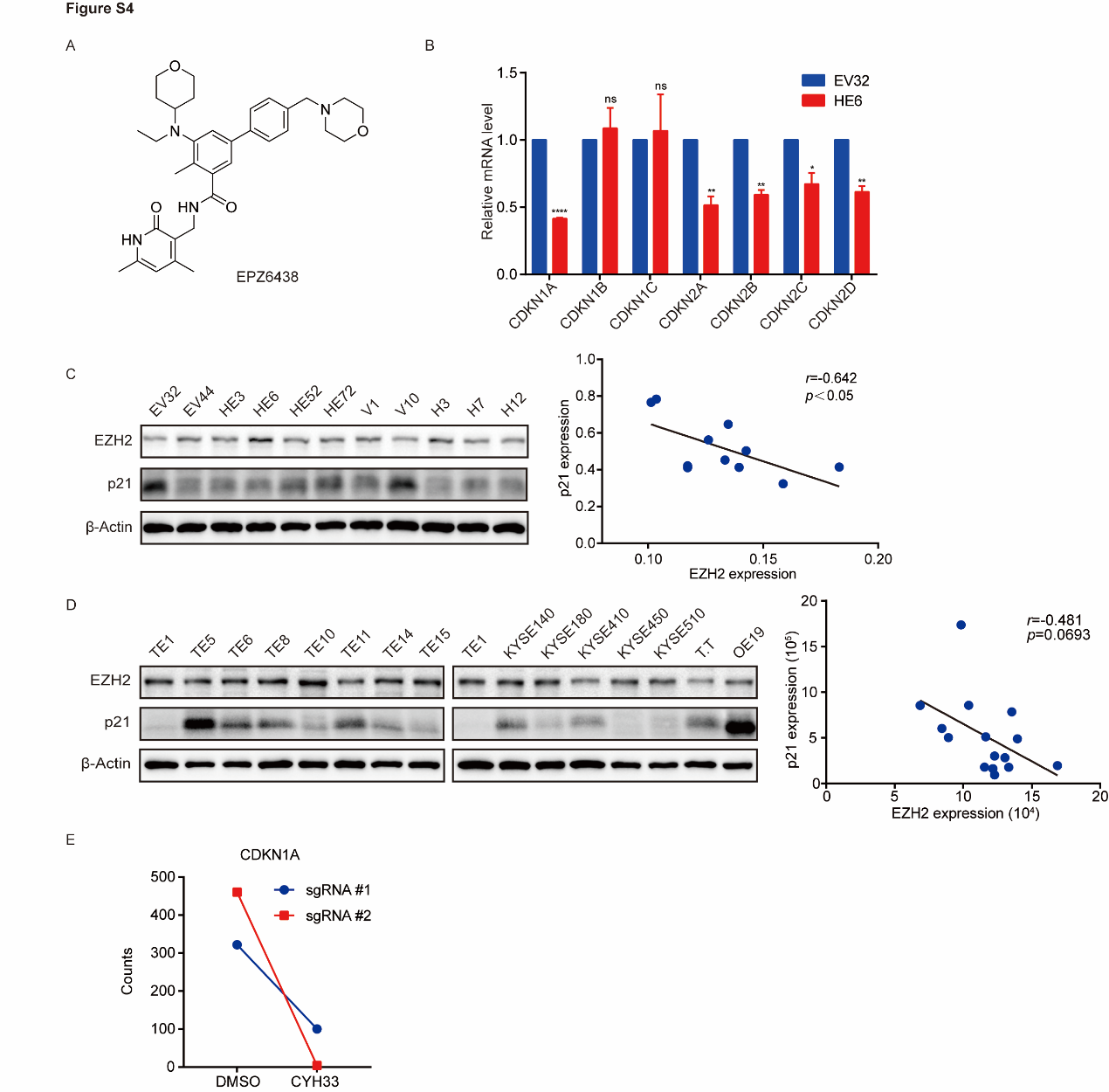


**FIGURE S4** Epigenetic repression of *CDKNIA* by EZH2 attenuated the activity of CYH33 against ESCC. (A) Structure of the EZH2 inhibitor EPZ6438. (B) HE6 cells stably expressing EZH2 or EV32 cells transfected with the empty vector were incubated with CYH33 (1 μM) for 24 h and total RNAs were extracted for quantitative PCR with primers targeting the indicated genes. Data are presented as mean ± SD from three independent experiments. Difference between two groups were analyzed by unpaired *t* test. ns, no significance. **p* < 0.05, ***p* < 0.01 and *****p* ＜ 0.0001. (C) A panel of clones were generated by transfecting KYSE510 with lentiviral plasmids expressing human EZH2 (HE or H) or empty vectors (EV or V). Protein levels of EZH2 and p21 in clones were detected by Western blot (*left*) and quantified by ImageJ. Pearson correlation analysis of the expression of EZH2 and p21 (*right*). (D) Protein levels of EZH2 and p21 in a panel of ESCC cell lines were detected by Western blot (*left*) and quantified by ImageJ. Pearson correlation analysis of the expression of EZH2 and p21 (*right*). (E) The counts of sgRNAs targeting *CDKN1A* in DMSO- and CYH33-treated cells of the CRISPR-SAM-based screening.


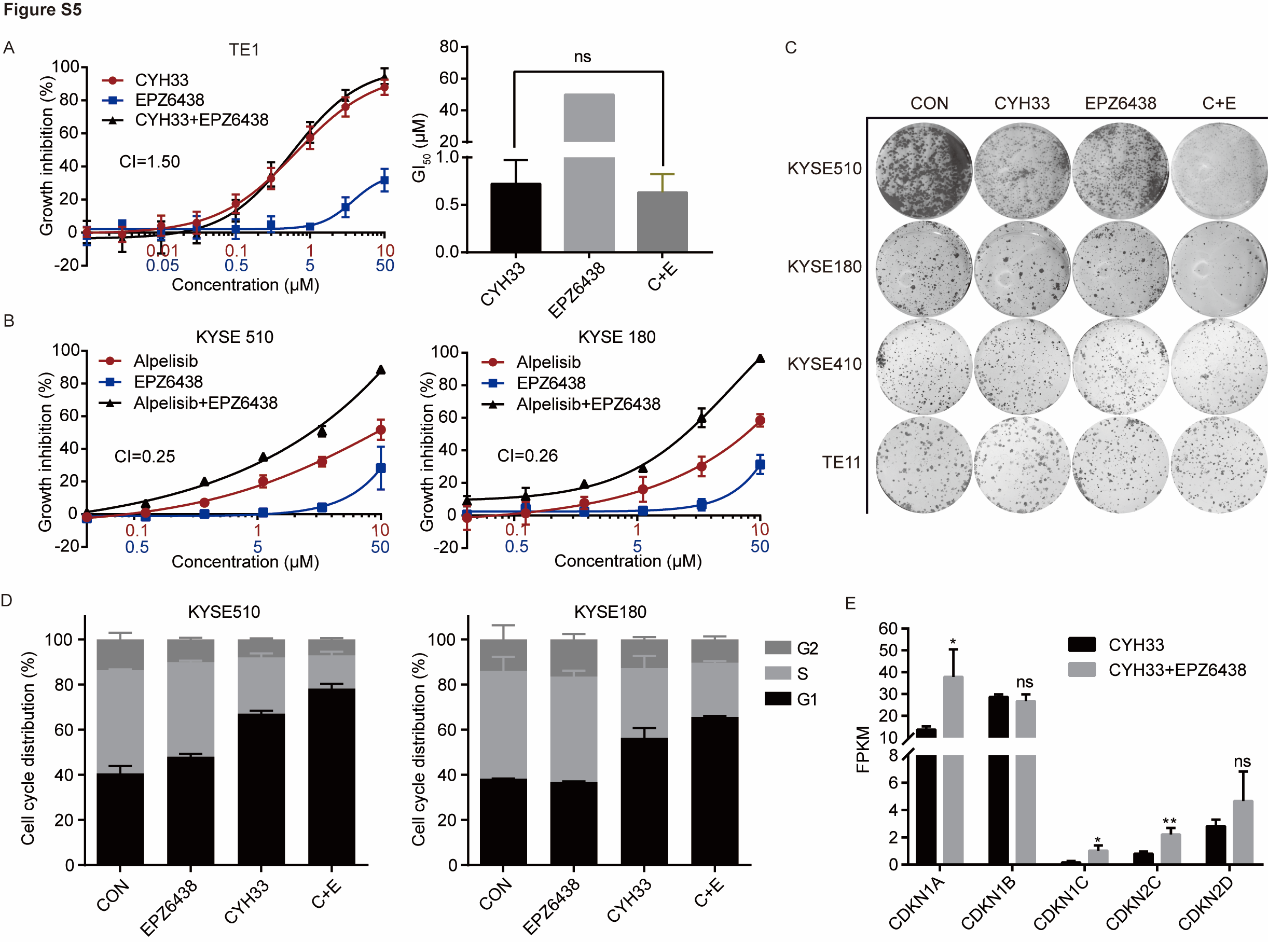


**FIGURE S5** The concomitant inhibition of PI3Kα and EZH2 displayed synergistic activity against ESCC cells. (A) RB-deficient TE1 cells were treated with CYH33 and EPZ6438 alone or concurrently for 5 d. Cell proliferation (*left*) and GI_50_s (*right*) were detected by SRB assay. Data shown are mean ± SD from three independent experiments. Difference between the indicated groups were determined by unpaired *t* test. ns, no significance. (B) KYSE510 and KYSE180 cells were incubated with alpelisib and EPZ6438 alone or concurrently for 5 d and cell proliferation were determined by SRB assay (*n* = 3). (C) Colony formation assay of ESCC cells upon treatment of CYH33 (0.1 μM) and EPZ6438 (5 μM) alone or concurrently for 10 d. (D) KYSE510 and KYSE180 cells were exposed to EPZ6438 (5 μM) for 48 h and then concurrently with CYH33 (0.1 μM) for additional 24 h. Cell cycle distribution was analyzed by flow cytometry. Data are presented as mean ± SD from three independent experiments. (E) The FPKM (Fragments Per Kilobase of transcript per Million mapped fragments) values of CKIs in cells treated with CYH33 (1 μM) alone or concurrently with EPZ6438 (5 μM) were determined by RNA sequencing. Difference between groups were analyzed by unpaired *t* test. ns, no significance. **p* < 0.05 and ***p* < 0.01.


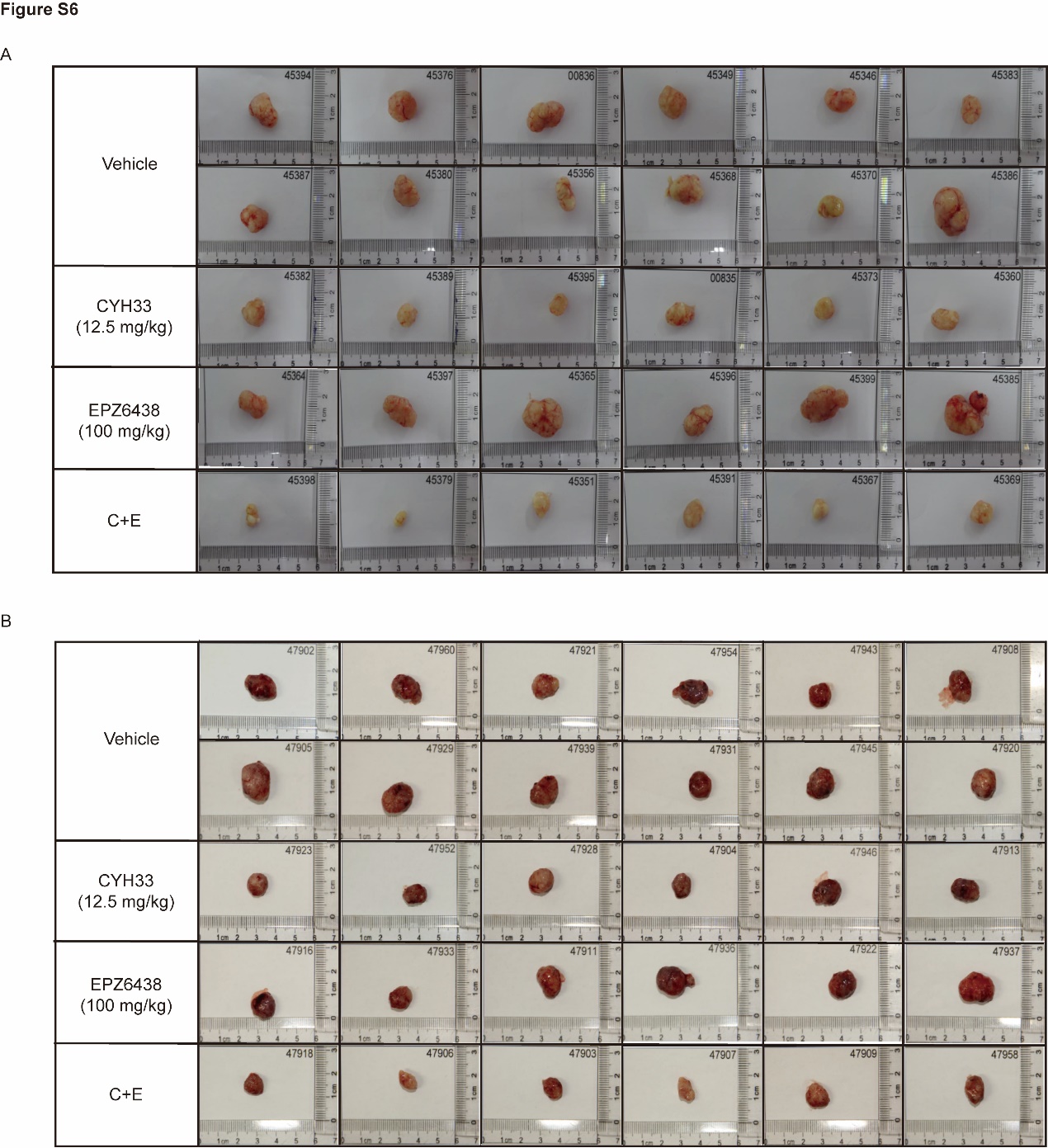


**FIGURE S6** EPZ6438 potentiated CYH33 to inhibit the growth of ESCC PDXs. (A, B) Images of tumor tissues collected from BALB/c nude mice bearing ESCC PDX ES-06-0003 (A) and ES-06-0016 (B) at the end of treatment.

**TABLE S1** The sequences of primers for the two-step PCR.

| Target name | Direction | 5’-3’ |
| --- | --- | --- |
| SAM-1 | Forward | GAGGGCCTATTTCCCATGATTCCTTCATATTTGCAT |
|  | Reverse | TTTTAAATGGCTAGAGACTTATCGAAAGCAGCGAGA |
| SAM-2 | Forward | GAAAGTATTTCGATTTCTTGGCTTTATATATCTTGTGG |
|  | Reverse | ATGTTGGCCAAGTTGATAACGGACTAGCC |

**TABLE S2** The sequences of siRNAs.

| Target name | 5’-3’ |
| --- | --- |
| EZH2 #1 | GGAUGGUACUUUCAUUGAATT |
| EZH2 #2 | CGGCUUCCCAAUAACAGUATT |
| EZH2 #3 | GAGGGAAAGUGUAUGAUAATT |
| NC | UUCUCCGAACGUGUCACGUTT |
| GAPDH | UGACCUCAACUACAUGGUUTT |

NC, negative control.

**TABLE S3** The sequences of primers for RT-qPCR.

| Gene | Direction | 5’-3’ |
| --- | --- | --- |
| EZH2 | Forward | AATCAGAGTACATGCGACTGAGA |
|  | Reverse | GCTGTATCCTTCGCTGTTTCC |
| CDKN1A | Forward | TGTCCGTCAGAACCCATGC |
|  | Reverse | AAAGTCGAAGTTCCATCGCTC |
| CDKN1B | Forward | TAATTGGGGCTCCGGCTAACT |
|  | Reverse | TGCAGGTCGCTTCCTTATTCC |
| CDKN1C | Forward | GCGGTGAGCCAATTTAGAGC |
|  | Reverse | TGCTACATGAACGGTCCCAG |
| CDKN2A | Forward | GGGTTTTCGTGGTTCACATCC |
|  | Reverse | CTAGACGCTGGCTCCTCAGTA |
| CDKN2B | Forward | CTGGACCTGGTGGCTACG |
|  | Reverse | ACATTGGAGTGAACGCATCG |
| CDKN2C | Forward | GGGACCTAGAGCAACTTACTAG |
|  | Reverse | CAAATCACAGGCGGTGTCC |
| CDKN2D | Forward | AGTCCAGTCCATGACGCAG |
|  | Reverse | ATCAGGCACGTTGACATCAGC |
| β-Actin | Forward | CATGTACGTTGCTATCCAGGC |
|  | Reverse | CTCCTTAATGTCACGCACGAT |

**TABLE S4** The sequences of primers for ChIP-qPCR.

| Gene | Direction | 5’-3’ |
| --- | --- | --- |
| CDKN1A-P1 | Forward | CCTCCTTCTTCAGGCTTGGG |
|  | Reverse | CAGGCAGCATAGGGATGGAG |
| CDKN1A-P2 | Forward | CTGCCTCTGCTCAATAATGTTCT |
|  | Reverse | GGAATTCACCTTCACACAGGC |

**TABLE S5** Correlation between EZH2 expression and the survival time grouped by clinicopathologic factors in Chinese ESCC patients

| **Variables** | **Number of cases** | | **Median survival time (months)** | | |
| --- | --- | --- | --- | --- | --- |
|  | **EZH2 high** | **EZH2 low** | **EZH2 high** | **EZH2 low** | ***p*^1^** |
| **Age** |  |  |  |  |  |
| ≤60 | 12 | 12 | 36.6 | 52.4 | 0.0198^*^ |
| ＞60 | 27 | 34 | 39.7 | 45.85 | 0.0472^*^ |
| **Gender** |  |  |  |  |  |
| Male | 33 | 38 | 36.6 | 47.9 | 0.0012^**^ |
| Female | 6 | 8 | 47.95 | 42.2 | 0.8922 |
| **AJCC^2^ Staging** |  |  |  |  |  |
| 0/Ⅰ | 16 | 8 | 37.9 | 43.3 | 0.3296 |
| Ⅱ | 14 | 26 | 37.85 | 52 | 0.0092^**^ |
| Ⅲ/Ⅳ | 9 | 12 | 36.8 | 36.5 | 0.3572 |
| **T^3^ status** |  |  |  |  |  |
| Tis/T1 | 4 | 2 | 39.05 | 55.4 | 0.3518 |
| T2 | 13 | 10 | 37.3 | 52.25 | 0.0171^*^ |
| T3/T4 | 22 | 34 | 37.05 | 45.7 | 0.0267^*^ |
| **N^4^ status** |  |  |  |  |  |
| N0 | 29 | 33 | 37.3 | 46.4 | 0.0271^*^ |
| N1 | 10 | 13 | 37.6 | 48.5 | 0.0242^*^ |
| **M^5^ status** |  |  |  |  |  |
| M0 | 37 | 42 | 38.4 | 48.6 | 0.0010^**^ |
| M1 | 2 | 4 | 23.6 | 27.5 | 0.3209 |
| **Therapy** |  |  |  |  |  |
| Radiotherapy | 37 | 41 | 38.4 | 48.7 | 0.0009^***^ |
| Chemoradiotherapy | 2 | 5 | 23.6 | 29.6 | 0.1865 |

^1^Log-rank test; ^2^AJCC, [American Joint Committee on Cancer](https://www.facs.org/quality-programs/cancer/ajcc); ^3^T, tumor; ^4^N, node; ^5^M, metastasis; **p* < 0.05, ***p* < 0.01 and *** *p* < 0.001.
